# Midazolam alleviates cellular senescence in SH‐SY5Y neuronal cells in Alzheimer's disease

**DOI:** 10.1002/brb3.2822

**Published:** 2022-11-28

**Authors:** Ping Wang, Peipei Wang, Hengfei Luan, Yong Wu, Ying Chen

**Affiliations:** ^1^ Department of Anesthesiology The First People's Hospital of Lianyungang Xuzhou Medical University Affiliated Hospital of Lianyungang Lianyungang Jiangsu China

**Keywords:** aging, Alzheimer's disease, Midazolam, oligomerized amyloid β, senescence

## Abstract

**Background:**

Alzheimer's disease (AD) impacts the daily life of aging people. Oligomerized amyloid β (Aβ)‐associated neuronal senescence is involved in the pathological mechanism of AD. Blockage of neuronal senescence has been considered an important strategy for the treatment of AD. Midazolam is a hypnotic‐sedative drug with pleiotropic properties.

**Aims:**

However, the effects of Midazolam in oligomerized Aβ_1.42_‐induced neurotoxicity have not been reported previously. Here, we investigate whether Midazolam possesses a beneficial effect against oligomerized Aβ_1.42_ in SH‐SY5Y neuronal cells.

**Materials and Methods:**

Cellular senescence was assessed using senescence‐associated β‐galactosidase staining. Telomerase activity was measured using the TeloTAGGG Telomerase PCR ELISA.

**Results:**

First, the lactate dehydrogenase release assay demonstrates that 10 and 20 µM are the optimal concentrations of Midazolam used for cell cultures. Senescence‐associated β‐galactosidase staining results indicate that exposure to oligomerized Aβ_1.42_ significantly increased cellular senescence of SH‐SY5Y cells, but it was significantly alleviated by Midazolam. Additionally, Midazolam restored the oligomerized Aβ_1.42_‐induced reduction of telomerase activity. Interestingly, we found that oligomerized Aβ_1.42_ remarkably reduced human telomerase reverse transcriptase (hTERT) gene expression but increased the telomeric repeat‐binding factor 2 (TERF2) expression. However, treatment with Midazolam reversed the effects of oligomerized Aβ_1.42_ on the hTERT and TERF2 gene expressions. Importantly, the presence of Midazolam attenuated Aβ_1.42_‐induced p53 and p21 expressions. Mechanistically, Midazolam repressed the level of cyclooxygenase‐2 (COX‐2) and the release of prostaglandin E2. Importantly, overexpression of COX‐2 abolished the impact of Midazolam against oligomerized Aβ_1.42_ in neuronal senescence.

**Conclusion:**

We conclude that the usage of Midazolam is a potential treatment strategy for AD.

## Graphical abstract

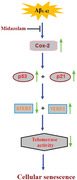

## 1 INTRODUCTION

Alzheimer's disease (AD) is a common central neurodegenerative disease and a significant inducer of cognitive dysfunction in the elderly (Lane et al., 2018). Declined cognitive function, memory loss, personality changes, and impaired normal social and emotional behaviors could be induced by the degeneration of neurons involved in learning and memory (Lane et al., 2018; Levenson et al., 2014; Sona et al., 2013). As Alois Alzheimer first proposed the concept of AD, many studies have been conducted to understand its mechanism (Takahashi et al., 2017). However, currently, an effective clinical treatment strategy for AD is rare. It has been projected that the number of patients affected by AD globally will reach 100 million if an effective drug is unavailable by 2050 (Hanger et al., 2009). Therefore, it is of great significance to explore its pathogenesis and treatment strategies. In recent years, studies have been conducted on the pathogenesis of AD, amongst which neurofibrillary tangles, nerve plaques, cortical neurons, and synaptic loss have been considered the main pathological features of AD (Terry et al., 1991). Deposition of amyloid β‐protein (Aβ) is considered to be a well‐established pathological mechanism of AD. It is decomposed from amyloid precursor protein (APP) (Sadigh‐Eteghad et al., 2015). APP is composed of nontoxic Aβ monomers, neurotoxic Aβ fibers, and polymerization of Aβ oligomers (Sardar Sinha et al., 2018). Normally, a dynamic balance between the production and clearance of Aβ is maintained (Shankar & Walsh, 2009). However, under aging or pathological conditions, the balance is disrupted (Harkany et al., 2000), leading to the accumulation of Aβ and the formation of senile plaques (Qiu et al., 2009). The accumulation of Aβ in the cerebral cortex is considered the main cause of AD (Masters et al., 1985). APP is a transmembrane protein that is expressed at a high level in the brain and metabolized in a fast and complex way (O'Brien & Wong, 2011). It can be cleaved by β secretase and γ secretase to produce Aβ_1–40_ and Aβ_1–42_ (Breydo et al., 2016). Recent studies have shown that cellular senescence induced by Aβ accumulation significantly facilitates the pathogenesis of AD (Shang et al., 2020; Zhang et al., 2019). Wang, Zheng, Yang, Wang, et al. (2021) showed that neuronal senescence was dramatically induced by Aβ_1–42_ oligomers but was greatly ameliorated by Agomelatine, implying a potential anti‐AD effect of Agomelatine. In addition, studies have shown that Olmesartan shows a potential anti‐AD effect by preventing neuronal aging induced by Aβ_1–42_ oligomers (Wang, Zheng, Yang, Zhou, et al., 2021). Therefore, an in‐depth study on the treatment strategies against Aβ_1–42_ oligomers‐induced neuronal senescence could be beneficial to exploring effective anti‐AD drugs.

**FIGURE 1:**
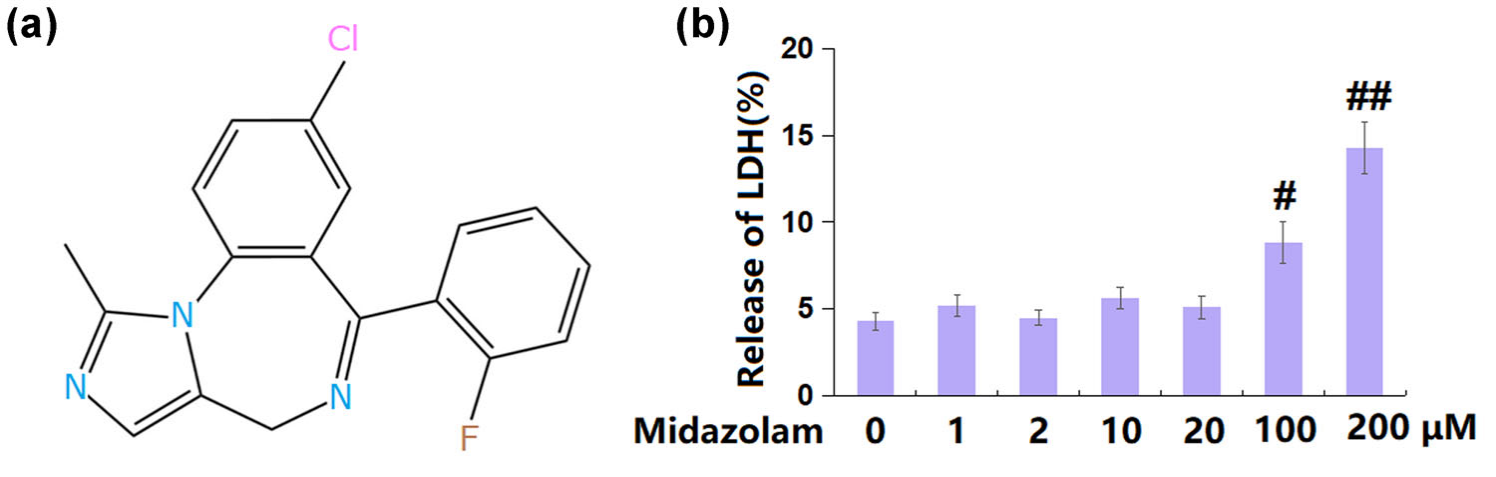
The effects of Midazolam on lactate dehydrogenase (LDH) release in SH‐SY5Y neuronal cells. Cells were stimulated with 1, 2, 10, 20, 100, and 200 μM Midazolam for 24 h. (a) Molecular structure of Midazolam; (b) release of LDH (#, ##, *p* < .05, .01 vs. vehicle group, *n* = 5–6)

Midazolam (Figure 1a), belonging to the benzodiazepine family, mainly acts on the limbic system and brainstem reticulation structure through binding with benzodiazepine receptors, showing anterograde amnesia, muscle relaxation, anti‐convulsion, hypnosis, sedation, and antianxiety effects (Redhair et al., 2020). By acting on γ‐aminobutyric acid type A receptor (GABAA) and transport factor protein (TSPO), Midazolam effectively facilitates the production of neurosteroids in CA1 vertebral neurons. Endogenous neurosteroids mainlyregulate depression and anxiety and also participate in the regulation of neuroprotection, nerve regeneration, feeding, sleep, convulsion, learning, and memory (Yuan Shi & Dong, 2020).

Based on these evidences, we hypothesize that Midazolam may have a protective effect on neuronal senescence induced by Aβ and could promote neuronal survival under neurodegenerative conditions. To validate this hypothesis, an Aβ_1–42_‐induced neuronal senescence culture model was established in vitro to investigate the potential regulatory function of Midazolam in AD. The underlying mechanism was also explored.

## 2 MATERIALS AND METHODS

### 2.1 Cell culture, treatment, and transduction

SH‐SY5Y neuronal cells were obtained from Procell (Wuhan, China) and cultured in MEM/F12 medium containing 15% FBS and 1% penicillin–streptomycin under the condition of 5% CO_2_ and 37°C. To obtain cyclooxygenase‐2 (COX‐2)‐overexpressed cells, SH‐SY5Y neuronal cells were infected with lentiviral COX‐2 for 2 days, and the efficacy of transfection was assessed with Western blot analysis. As described before (Guzmán et al., 2020), cells were stimulated with oligomerized Aβ_1–42_ (5 μM) with or without Midazolam (10, 20 μM) for 7 days.

### 2.2 Lactate dehydrogenase (LDH) release

SH‐SY5Y neuronal cells were plated in a 6‐well plate. The confluent cells were stimulated with 1, 2, 10, 20, 100, and 200 μM Midazolam for 24 h. To obtain the lactate dehydrogenase (LDH) level from cell culture, 120 μl cell supernatant was loaded into a 96‐well plate, then 60 μl LDH solution was added to each well. After incubating for half an hour in the dark, the absorbance value of each well was detected at 490 nm. The detected optical density value of each well was subtracted from that of the blank control well, and then the absorbance value of each group was standardized after normalization with the control group.

### 2.3 Senescence‐associated β‐galactosidase (SA‐β‐gal) staining

Cells were rinsed with 2 ml PBS, and then 1 ml fixing solution was added to each well, followed by being fixed for 15 min. After washing with 2 ml PBS, 1 ml senescence‐associated β‐galactosidase (SA‐β‐gal) staining solution was introduced into the wells. The culture plate was placed in an incubator with a constant temperature of 37°C without CO_2_ and incubated for 12–15 h, followed by sealing to prevent evaporation of the dyeing solution in the culture plate. The number of positive cells was checked under the microscope (Leica, Germany).

### 2.4 The telomerase activity

We measured the telomerase activity followed by the method described before (Mookherjee et al., 2007). In brief, the 3‐((3‐cholamidopropyl) dimethylammonio)‐1‐propanesulfonate buffer was used to lyse SH‐SY5Y cells for 30 min on the ice. Then the lysate was centrifuged at 15,000 *g* for 90 min. Isolated protein was quantified using the bicinchoninic acid (BCA) method, and the TeloTAGGG Telomerase PCR ELISA Plus Kit (Roche, Switzerland) was then utilized to detect the activity of telomerase. Subsequently, the telomeric repeat amplification protocol–PCR reaction system was introduced together with samples and primers. Lastly, RT‐PCR was used to perform the quantification.

### 2.5 Real‐time PCR

Briefly, the TRIzol reagent was used to isolate RNAs from SH‐SY5Y neuronal cells, followed by transcribing into cDNA with a HiScript II Q RT SuperMix for qPCR (+gDNA wiper) (Vazyme, China). Subsequently, the 2 × SYBR Green PCR Master Mix (Lifeint, China) was used to perform the PCR reaction with specific primers to determine the gene levels. To monitor the gene expression, the relative RNA level was presented using the 2^−ΔΔ^ *^Ct^* method after normalization with the expression of glyceraldehyde 3 phosphate dehydrogenase (GAPDH). The following primers were used in this study: human telomerase reverse transcriptase (HTERT), forward, 5′‐GCCGATTGTGAACATGGACTACG‐3′, reverse, 5′‐GCTCGTAGTTGAGCACGCTGAA‐3′; telomeric repeat‐binding factor 2 (TERF2), forward, 5′‐GTGGAAAAGCCACCCAGAGAAC‐3′, reverse, 5′‐TGCAAAGGCTGCCTCAGAATCC‐3′; p53, forward, 5′‐CCTCAGCATCTTATCCGAGTGG‐3′, reverse, 5′‐TGGATGGTGGTACAGTCAGAGC‐3′; p21, forward, 5′‐AGGTGGACCTGGAGACTCTCAG−3′, reverse, 5′‐TCCTCTTGGAGAAGATCAGCCG−3′; GAPDH, forward, 5′‐GTCTCCTCTGACTTCAACAGCG‐3′, reverse, 5′‐ACCACCCTGTTGCTGTAGCCAA‐3′.

### 2.6 Western blot analysis

Cells in each group were lysed with lysis buffer, and the protein concentration was determined with the BCA method. The same amount of protein (20 μg) was added to the prepared gel, and electrophoresis was performed at 150 V at a constant voltage for 60 min. After electrophoresis, proteins were transferred to the polyvinylidene fluoride (PVDF) membrane, which was quickly put into 1 × TBST for rinsing (Shi et al., 2013). The primary antibody against p53 (1:1000, Abcam, UK), p21 (1:2000, Abcam, UK), COX‐2 (1:800, Abcam, UK), or β‐actin (1:10,000, SolelyBio, China) was added and incubated at 4°C overnight. The second antibody was then added and incubated at 25°C for 2 h. The luminescent solutions A and B were mixed at a ratio of 1:1 and coated on the surface of the PVDF membrane. Finally, Bio‐Rad Quantity One software was used for the quantitative analysis of bands.

### 2.7 Enzyme‐linked immunosorbent assay (ELISA)

The cell supernatant was centrifuged at 4°C for 10 min at 1000 *g* to remove the particles and polymer. The standard dilution and the sample to be tested were added to each well in a 96‐well plate, respectively. The biotin‐labeled antibody was subsequently introduced, followed by incubation at 37°C for 1 h. After removing the liquid, horseradish peroxidase solution was added and mixed gently, followed by 30 min incubation at 37°C. Then, substrates A and substrate B were added to each well to react at 37°C for 15 min in the light, followed by the immediate reaction with a 50 μl stop solution. Lastly, the absorbance of each well at 450 nm wavelength was determined with a microplate reader (Thermo Fisher, USA).

### 2.8 Statistical analysis

All the experiments have been repeated at least three times. Data were presented as mean ± standard deviation and analyzed utilizing the GraphPad Prism software (GraphPad, USA). The statistical analysis was performed with the one‐way analysis of variance method using the Tukey postK hoc test. *p* < .05 was considered a significant difference.

## 3 RESULTS

In this study, we investigated the beneficial effects of Midazolam in oligomerized Aβ_1–42_‐treated SH‐SY5Y neuronal cells. We found that 10 and 20 μM were the two safe concentrations of Midazolam not threatening cytotoxicity in cell cultures. Moreover, Midazolam significantly alleviated senescence progression, including the increase in telomerase activity and the reduction of p53 and p21. Mechanistically, Midazolam repressed COX‐2 to alleviate Aβ_1–42_‐induced neuronal senescence.

### 3.1 The effects of Midazolam on LDH release in SH‐SY5Y neuronal cells

SH‐SY5Y neuronal cells were stimulated with 1, 2, 10, 20, 100, and 200 μM Midazolam for 24 h, followed by checking for its toxicity on SH‐SY5Y neuronal cells. The release of LDH (Figure 1b) was maintained at around 5% as the concentration increased from 1 to 20 μM, but it was greatly elevated to 8.8% and 14.3% in 100 and 200 μM Midazolam‐treated SH‐SY5Y neuronal cells, respectively. Therefore, 10 and 20 μM were used in the subsequent experiments.

### 3.2 Midazolam ameliorated oligomerized Aβ_1–42_‐induced cellular senescence of SH‐SY5Y neuronal cells

**FIGURE 2:**
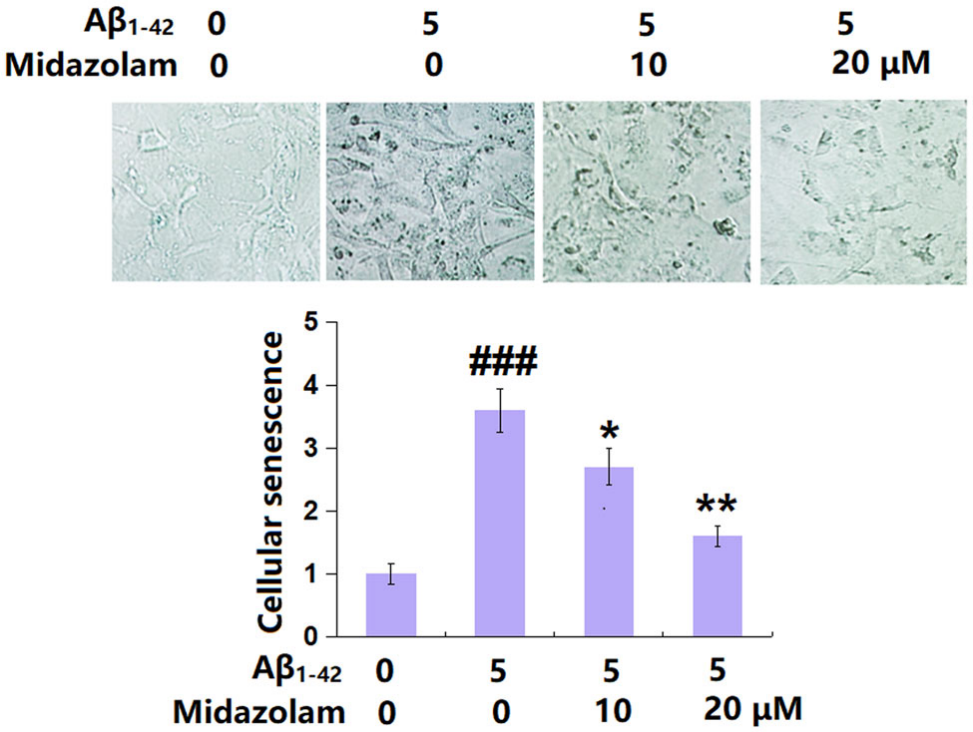
Midazolam ameliorated oligomerized Aβ_1–42_‐induced cellular senescence of SH‐SY5Y neuronal cells. Cells were stimulated with oligomerized Aβ_1–42_ (5 μM) with or without Midazolam (10 and 20 μM) for 7 days. Cellular senescence was assayed using senescence‐associated β‐galactosidase (SA‐β‐gal) staining (###, *p* < .005 vs. vehicle group; *, **, *p* < .05, .01 vs. oligomerized Aβ_1–42_ group, *n* = 5–6).

Cells were stimulated with oligomerized Aβ_1–42_ (5 μM) with or without Midazolam (10, 20 μM) for 7 days, followed by evaluating cellular senescence. We found that the number of SA‐β‐gal staining positive cells (Figure 2) was greatly increased in Aβ_1–42_‐stimulated cells but extremely reduced by 10 and 20 μM Midazolam, suggesting its inhibitory effect against Aβ_1–42_‐induced senescence in SH‐SY5Y neuronal cells.

### 3.3 Midazolam attenuated oligomerized Aβ_1–42_‐induced reduction of telomerase activity

**FIGURE 3:**
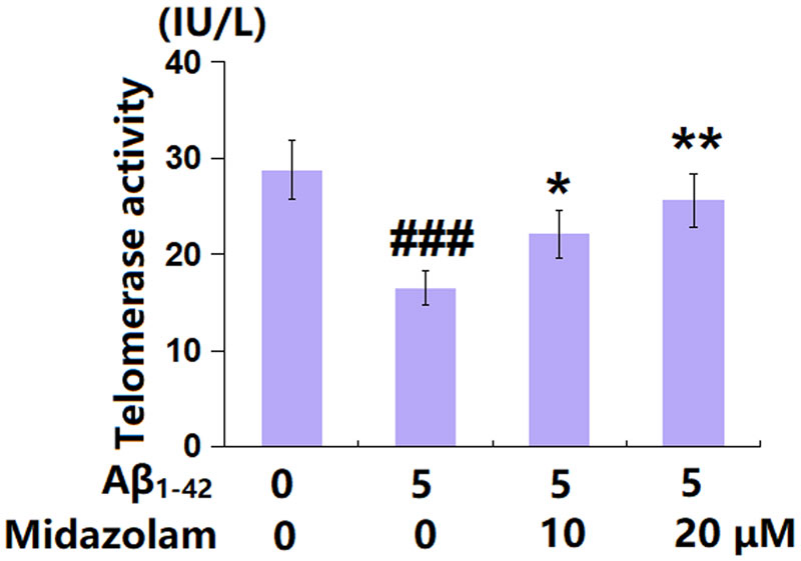
Midazolam attenuated oligomerized Aβ_1–42_‐induced reduction of telomerase activity in SH‐SY5Y cells. Cells were stimulated with oligomerized Aβ_1–42_ (5 μM) with or without Midazolam (10 and 20 μM) for 7 days. Telomerase activity was measured (###, *p* < .005 vs. vehicle group; *, **, *p* < .05, .01 vs. oligomerized Aβ_1–42_ group, *n* = 5–6).

Furthermore, the telomerase activity in each group was evaluated to confirm the effect of Midazolam on cellular senescence. The telomerase activity (Figure 3) in SH‐SY5Y cells was decreased from 28.8 to 16.5 IU/L by stimulation with Aβ_1–42_ but was elevated to 22.1 and 25.6 IU/L by 10 and 20 μM Midazolam, respectively, confirming its inhibitory effect against Aβ_1–42_‐induced senescence in SH‐SY5Y neuronal cells.

### 3.4 The effects of Midazolam on the hTERT and TERF2 expressions in Aβ_1–42_‐challenged SH‐SY5Y cells

**FIGURE 4:**
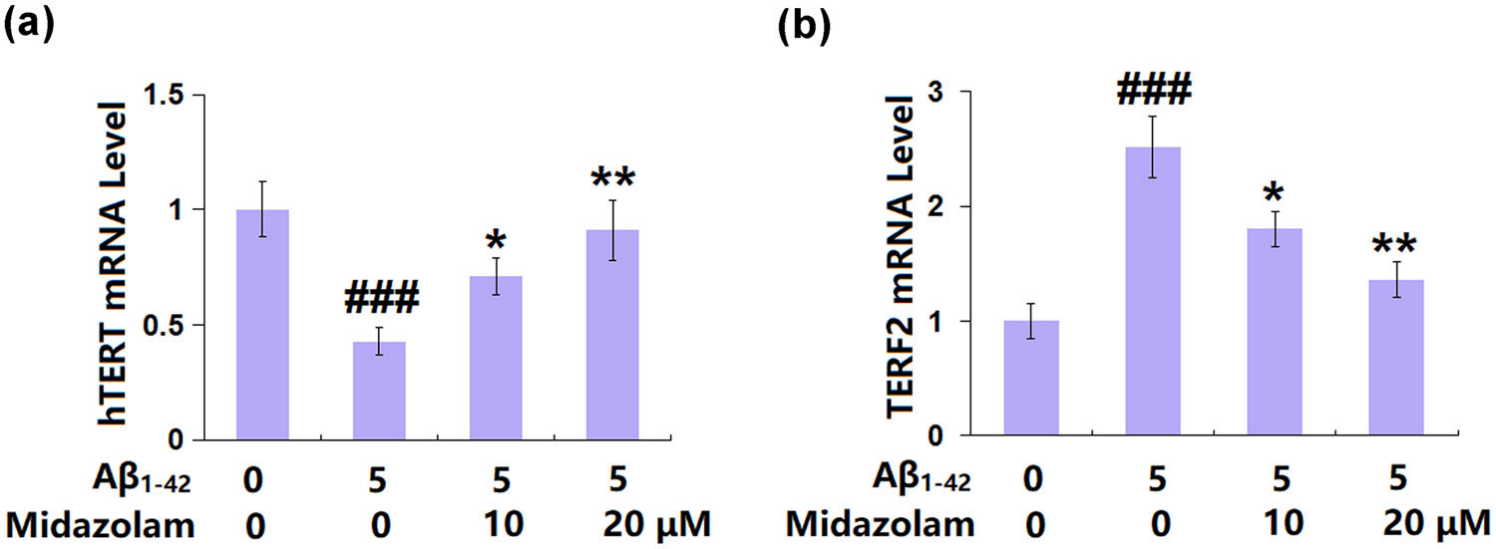
The effects of Midazolam on human telomerase reverse transcriptase (hTERT) and telomeric repeat‐binding factor 2 (TERF2) expressions in Aβ_1–42_‐challenged SH‐SY5Y cells. Cells were stimulated with oligomerized Aβ_1–42_ (5 μM) with or without Midazolam (10, 20 μM) for 7 days. (a) mRNA expression of hTERT; (b) mRNA of TERF2 (###, *p* < .005 vs. vehicle group; *, **, *p* < .05, .01 vs. oligomerized Aβ_1–42_ group, *n* = 5–6)

hTERT and TERF2 are important factors regulating telomere shortening (Darvishi & Saadat, 2021). We found that hTERT in SH‐SY5Y cells (Figure 4) was dramatically downregulated by Aβ_1–42_ but was significantly rescued by 10 and 20 μM Midazolam. In contrast, the oligomerized Aβ_1–42_‐induced increase in TERF2 was mitigated by Midazolam, implying that its regulatory effect on telomerase activity might be mediated by hTERT and TERF2.

### 3.5 Midazolam‐reduced p53 and p21 expression in Aβ_1–42_‐challenged SH‐SY5Y cells

**FIGURE 5:**
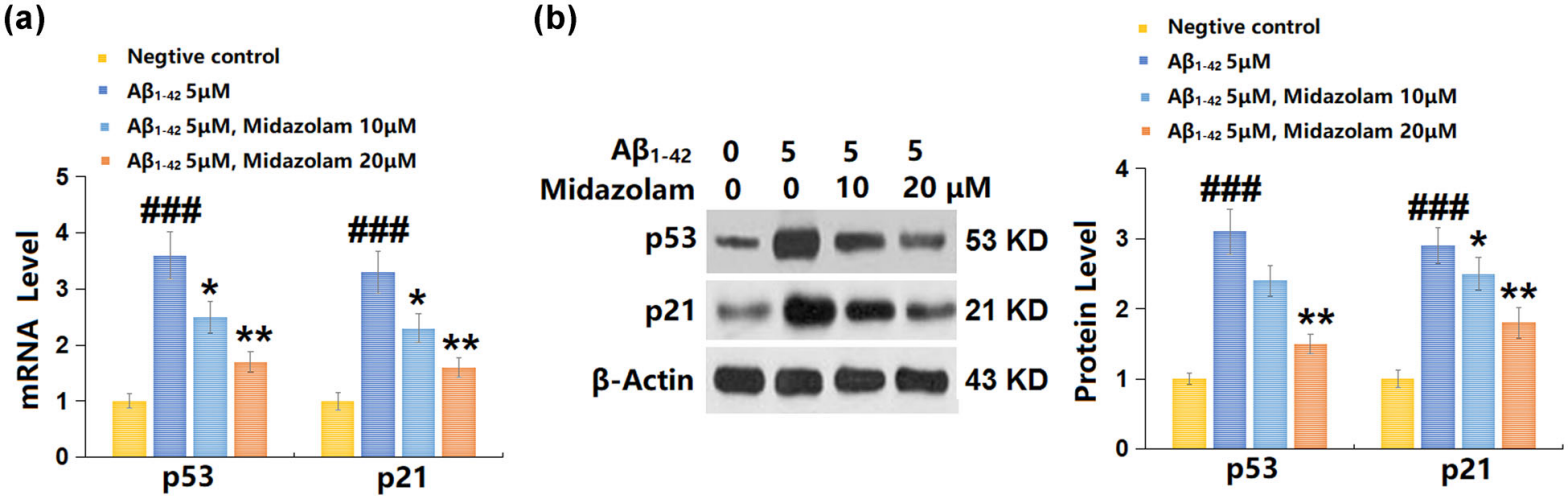
Midazolam‐reduced p53 and p21 expression in Aβ_1–42_‐challenged SH‐SY5Y cells. Cells were stimulated with oligomerized Aβ_1–42_ (5 μM) with or without Midazolam (10, 20 μM) for 24 h. (a) mRNA of p53 and p21; (b) protein of p53 and p21 (###, *p* < .005 vs. vehicle group; *, **, *p* < .05, .01 vs. oligomerized Aβ_1–42_ group, *n* = 5–6)

P53 and p21 are two iconic biomarkers for cellular senescence (Chen et al., 2020), and the expressions of which were further evaluated. The expression levels of p53 and p21 (Figure 5a,b) were extremely promoted in Aβ_1–42_‐challenged SH‐SY5Y cells, which were greatly reversed by 10 and 20 μM Midazolam, implying that its regulatory effect on cellular senescence might be mediated by p21 and p53.

### 3.6 Midazolam repressed the COX‐2 level and the release of PGE_2_ in Aβ_1–42_‐challenged SH‐SY5Y cells

**FIGURE 6:**
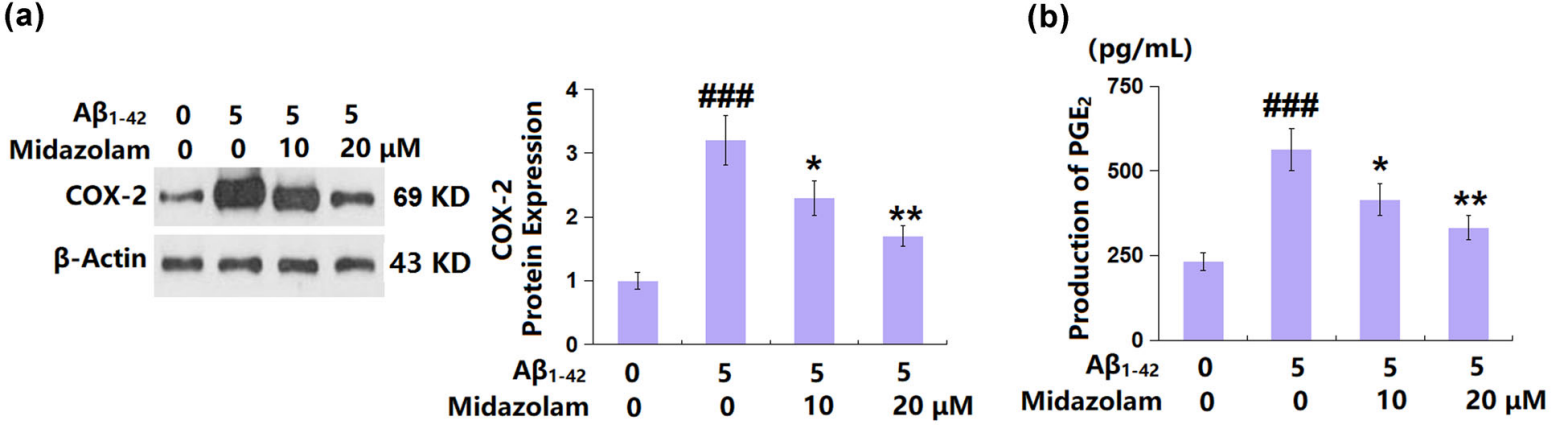
Midazolam‐reduced the expression of cyclooxygenase‐2 (COX‐2) and the production of prostaglandin E2 (PGE_2_) in Aβ_1–42_‐challenged SH‐SY5Y cells. Cells were stimulated with oligomerized Aβ_1–42_ (5 μM) with or without Midazolam (10, 20 μM) for 24 h. (a) Expression of COX‐2; (b) production of PGE_2_ (###, *p* < .005 vs. vehicle group; *, **, *p* < .05, .01 vs. oligomerized Aβ_1–42_ group, *n* = 5–6)

The COX‐2/PGE_2_ (prostaglandin E2) axis is involved in mediating cellular senescence (Goncalves et al., 2021). COX‐2 (Figure 6a) was dramatically upregulated in Aβ_1–42_‐challenged SH‐SY5Y cells but was greatly rescued by 10 and 20 μM Midazolam. Furthermore, the production of PGE_2_ (Figure 6b) was promoted from 231.5 to 563.2 pg/ml by Aβ_1–42_ then reduced to 415.6 and 332.7 pg/ml by 10 and 20 μM Midazolam, respectively. These data suggest that the activated COX‐2/PGE_2_ axis in Aβ_1–42_‐challenged SH‐SY5Y cells was suppressed by Midazolam.

### 3.7 Overexpression of COX‐2 abolished the protective effects of Midazolam against Aβ_1–42_ in SH‐SY5Y cells

**FIGURE 7:**
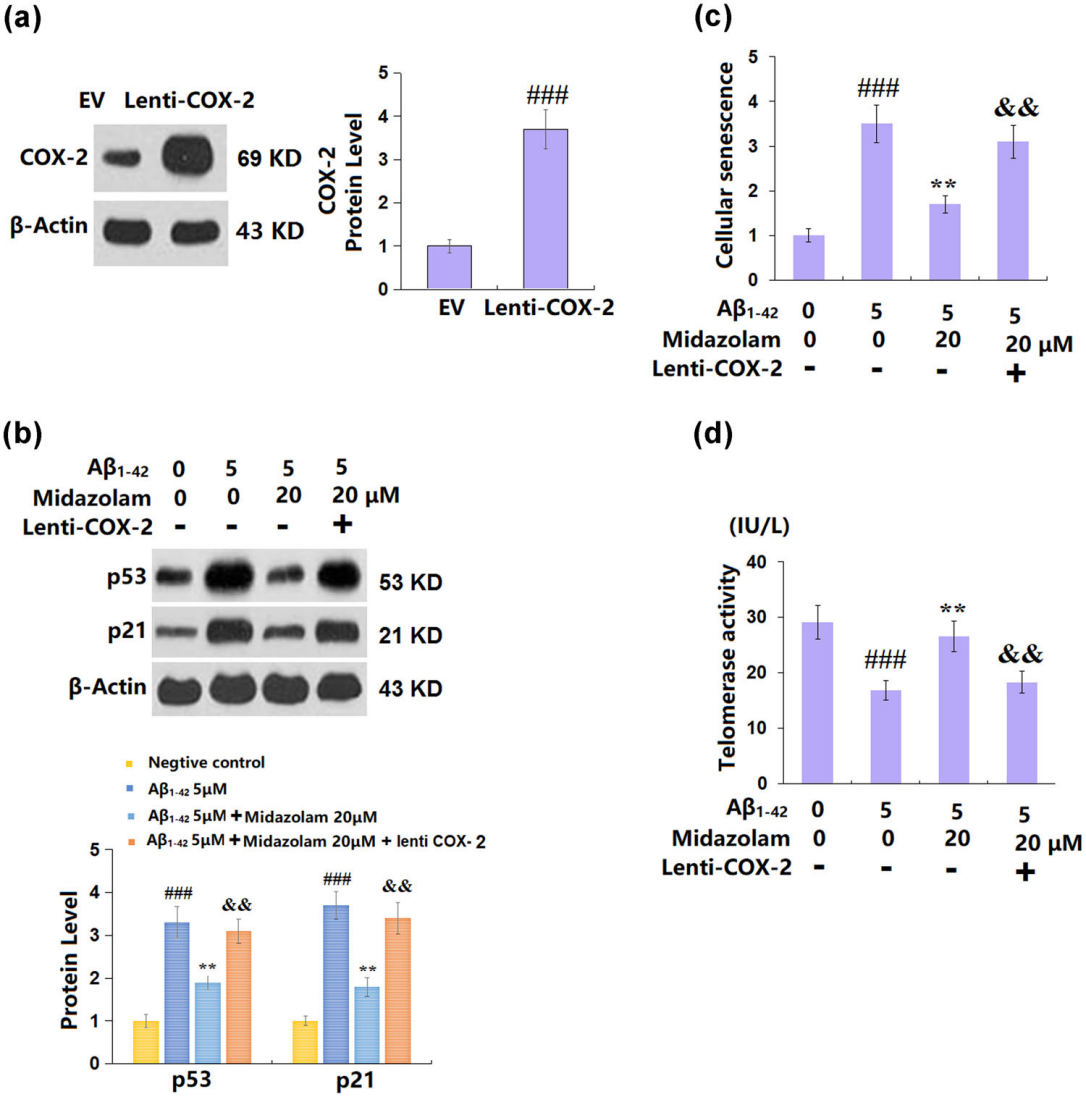
Overexpression of cyclooxygenase‐2 (COX‐2) abolished the protective effects of Midazolam against Aβ_1–42_ in SH‐SY5Y cells. Cells were transduced with lentiviral COX‐2, followed by stimulation with Midazolam (20 μM) with oligomerized Aβ_1–42_ (5 μM) for 7 days. (a) Real‐time PCR analysis revealed successful overexpression of COX‐2; (b) expression of p53 and p21; (c) cellular senescence; (d) telomerase activity (###, *p* < .005 vs. vehicle group; **, *p* < .01 vs. oligomerized Aβ_1–42_ group; &&, *p* < .01 vs. oligomerized Aβ_1–42_ + Midazolam group, *n* = 5–6)

To identify the function of COX‐2 in the regulation of Midazolam against cellular senescence induced by Aβ_1–42_, COX‐2 overexpressed SH‐SY5Y cells were established. Cells were transfected with lentiviral COX‐2, followed by stimulation with Midazolam (20 μM) with oligomerized Aβ_1–42_ (5 μM) for 7 days. The efficacy of transfection was verified by real‐time PCR (Figure 7a). The increased levels of p53 and p21 in Aβ_1–42_‐challenged SH‐SY5Y cells (Figure 7b) were greatly repressed by Midazolam, which was reversed by the overexpression of COX‐2. Importantly, the protective effects of Midazolam in cellular senescence (Figure 7c) and telomerase activity (Figure 7d) were abolished by overexpressing COX‐2, suggesting the protective effects of Midazolam against Aβ_1–42_ in SH‐SY5Y cells might be associated with the downregulation of COX‐2.

## 4 DISCUSSION

As a cellular stress response, cellular senescence contributes to the declined proliferation in response to growth factors or mitogens (Kuilman et al., 2010). Senescent cells show morphological changes such as cell flattening and swelling, increased β‐gal activity, decreased replication ability, cell cycle arrest, elevated levels of p53 and p21, and formation of senescence‐related heterochromatin foci (Qian & Chen, 2013). p21 is the classical target gene of p53 and a cell cycle inhibitor (Qian & Chen, 2013). Activated p21 specifically binds to the cyclin/cyclin kinase complex during the G1 phase of the cell cycle to inhibit its kinase activity. By interacting with proliferating cell nuclear antigen, p21 suppresses DNA replication (Mansilla et al., 2020). Under DNA damage, hypoxia, and oncogene activation stimuli, the regulation of p53 on the G1 monitoring site in the cell cycle is mediated by p21. Cell senescence is closely related to cell cycle arrest, suggesting that p21 plays an essential role in cell senescence. Studies have shown that p21 is significantly upregulated in replicative aging. In p21‐knockout human microvascular endothelial cells, the RAS‐mediated cellular senescence was abolished entirely (Borgdorff et al., 2010). We found that cellular senescence was significantly triggered in SH‐SY5Y cells by the stimulation with Aβ_1–42_, accompanied by the upregulation of p21 and p53, in accordance with the observations reported by Wang (Wang, Zheng, Yang, Wang, et al., 2021). After the incubation with 10 and 20 μM Midazolam, cellular senescence was dramatically alleviated, accompanied by the downregulation of p21 and p53, implying an inhibitory property of Midazolam against Aβ_1–42_‐induced senescence in neurons.

Human telomerase consists of two core components, the RNA component (human telomerase RNA) and the catalytic protein subunit (hTERT) (Lingner et al., 1997; Smith et al., 1997). In normal somatic cells, telomerase shortening often results in cell growth arrest or programmed death. In immortalized cells and tumor cells, telomerase activity is upregulated to maintain cell growth potential (Shay & Wright, 2011; Wang et al., 2010). It is reported that telomerase activity is elevated significantly in advanced tumor cells, whereas in normal cells, telomerase levels are maintained at low or almost undetectable levels. hTERT is a core component of telomerase, a characteristic indicator of telomerase activity, and can be used as a monitoring indicator in tumor cells (Redon et al., 2010; Ruden & Puri, 2013). TERF2 is a component of shelterin and binds to telomere DNA through the carboxyl‐terminal to play an important role in maintaining the structural stability of telomere DNA. It prevents end‐to‐end fusion of chromosomes and participates in DNA damage response, which is another marker of telomerase activity (Benhamou et al., 2016). Dramatically declined telomerase activity was observed in Aβ_1–42_‐challenged SH‐SY5Y cells in the present study, consistent with the report by Wang (Wang, Zheng, Yang, Wang, et al., 2021). Moreover, dysregulated hTERT and TERF2 were observed in Aβ_1–42_‐challenged SH‐SY5Y cells in the present study, which further confirmed the effect of Aβ_1–42_ on the telomerase activity in SH‐SY5Y cells. After the treatment with 10 and 20 μM Midazolam, the telomerase activity was rescued, accompanied by the restoration of hTERT and TERF2, implying a repair function of Midazolam on the impaired telomerase activity in Aβ_1–42_‐challenged neurons.

Recently, it is reported that the COX‐2/PGE_2_ axis critically regulates cellular senescence by shaping the composition of the senescence‐associated secretory phenotype and increasing senescence surveillance (Goncalves et al., 2021). We found that the activated COX‐2/PGE_2_ axis in Aβ_1–42_‐challenged SH‐SY5Y cells was profoundly repressed by Midazolam. Furthermore, the inhibitory effect of Midazolam on cellular senescence in Aβ_1–42_‐challenged SH‐SY5Y cells was largely rescued by the overexpression of COX‐2, suggesting that it might mediate the impact of Midazolam on cellular senescence in Aβ_1–42_‐challenged neurons. However, the effects of COX‐2 on p53 and p21 expression are still unknown. A further study will explore the underlying mechanism.

The limitation of the current study has to be addressed. First, the molecular mechanism of Midazolam in neurons remains to be fully understood in the setting of amyloid accumulation. Midazolam activates GABA_A_ receptors, promotes the binding of GABA to receptor A, and facilitates the opening of chloride channels to increase the inward chloride influx. In immature neurons, Midazolam altered the mRNA GABA receptor subunit expressions (Sinner et al., 2019). The altered expression of GABA receptor mostly like could be involved in certain aspects of neuronal regulation. In the mature neurons, this type of regulation remains to be identified. So far, it is unclear whether GABA receptor expression is associated with its neuroprotective effect. Second, all the current tests were performed in vitro, and the efficacy of the neuroprotection by Midazolam in the setting of amyloid accumulation has to be validated in AD animal model.

## 5 CONCLUSION

In summary, our data reveal that the hypnotic‐sedative drug Midazolam had a beneficial effect on alleviating cellular senescence in brain neuronal cells. Our study confirms that Midazolam ameliorated Aβ_1–42_‐induced neuronal senescence by suppressing the COX‐2/PGE_2_ axis. These data suggest that Midazolam possesses neuroprotective effects in the setting of Aβ challenge in vitro; however, its efficacy remains to be investigated in an AD model.

## CONFLICT OF INTEREST

The authors declare that there is no conflict of interest that could be perceived as prejudicing the impartiality of the research reported.

## CONSENT TO PUBLICATION

All the authors agreed to publish this article.

### Peer Review

The peer review history for this article is available at: https://publons.com/publon/10.1002/brb3.2822

## Data Availability

The data and materials of this study are available upon reasonable request from the corresponding authors.
